# An Essay Towards a History of Midwifery

**Published:** 1842-07

**Authors:** 


### Art. VI.

*Versuch einer Geschichte der Geburtshülfe.* Von Dr. E. C. J. Von Siebold, &c. Erster Band.—*Berlin*, 1839. 8vo, pp. 384.

*An Essay towards a History of Midwifery.* By Dr. E. C. J. Von Siebold, &c. Vol. I.—*Berlin*, 1839.

The brief and imperfect sketches of the history of the obstetric art, furnished by Smellie, Leake, and Denman, continue to be the only records in our language of the progress of this branch of medicine. The subject has attracted much more attention in France, to which country we are indebted for several historical sketches of considerable value, though they are all inferior to a work which was published at Göttingen, at the close of last century, by the famous Osiander, who then occupied the midwifery chair in that university. This "History of Midwifery," which appeared in the year 1799, contains a store of materials, which has been much resorted to down to the present time. Its merits are very great, but from them there is one very serious drawback, namely, the asperity and unfairness with which the author criticises the labours of all whose opinions are at variance with his own. M. von Siebold, with more than equal learning and research, has brought to the task a much sounder and more impartial judgment and better taste, while he is altogether free from that spirit of anecdote-mongering in which his garrulous predecessor so largely indulged.

The history of the subject is divided, by Von Siebold, into three periods: the ancient, the middle, and the modern. The first extends from the earliest times to the cultivation of medicine by the Arabs, about the end of the seventh century; the second reaches to the end of the seventeenth century, an epoch marked by the invention of the forceps, and the publication of the Novum Lumen of Deventer; and the third brings us down to the present day. Each of these periods is distinguished by a peculiar character. In ancient times, the practice of midwifery was exclusively in the hands of females, the physician being called in only in consultation, and chiefly in cases where benefit was anticipated from pharmaceutical measures. Operative midwifery was then characterized by disregard of the life of the child; and perforation and embryotomy were almost the only operations to which recourse was had. In the middle period, midwifery was regarded merely as a branch of surgery; hence its practice was purely mechanical. The last period is distinguished by the employment of the forceps, by great improvements in the whole of operative midwifery, by a juster appreciation of the powers of nature, and of the circumstances calling for the interference of art. M. von Siebold further subdivides each of these periods by a judicious selection of epochs, the first into three, the second into four, the last into two eras. We will hastily review the characteristic features of the first five of these periods, which are treated of in the present volume; the remaining four, which embrace the history of midwifery since the year 1532, will form the subject of the second volume.

*First period.* From the earliest times to Hippocrates, or to the end of the fifth century before the christian era.

After a few preliminary remarks on the difficulties of investigation, into the origin of any of the arts or sciences, M. von Siebold observes that midwifery in its simplest form must have existed long before any attempts were made to heal diseases. In most uncivilized nations, and even in many parts of the east, some female relative attends upon a woman when in labour. This was doubtless the practice in early times, and long after the attendance upon women in labour had become a distinct occupation, the office was undertaken by none but females. The bible contains frequent mention of midwives, but says nothing which could lead to the supposition that the interference of men was permitted under any circumstances. Nor is there any evidence to show, as Osiander imagined, that the Egyptian priests ever attended upon women in labour, while the condition of medicine among the Egyptians, and their known ignorance of surgery, preclude such a supposition. Further, the mythology of Egypt and Greece tends to show that women were the sole practitioners of midwifery in those countries, since the deities who presided over pregnancy and childbirth were all females. The two principal of these goddesses were Isis and Eileithyia; the former was implored by Egyptian women, the latter by those of Greece, to preserve them during labour, and to send them a safe and speedy delivery. The actual assistance rendered to women during parturition was small, and probably did not exceed placing the patient in a convenient position, receiving the child when born, or extracting it if not quite expelled, separating and securing the navel string, and perhaps withdrawing the placenta by traction at the umbilical cord. (pp. 1-69.)

*Second period.* From Hippocrates to the decline of science after Galen, or to the commencement of the third century after the christian era.

The age of fable and tradition past, we come to the first written rules for the practice of the obstetric art. The observations relating to the subject in the true Hippocratic books are but very few, and show that women had the practice of midwifery entirely in their hands, and that the help of the physician was sought only in some extraordinary difficulty.

He could, however, render but little assistance, since he was deprived of every opportunity for watching the process of natural labour. This is fully proved by various passages in the spurious Hippocratic books which treat much more fully on midwifery than those which are attributed to Hippocrates himself. All feet presentations were regarded as extremely hazardous; and whenever the child was living, attempts were made to bring down the head, since in none but head presentations was delivery supposed to be practicable. The manner in which this practice is insisted on in all the oldest medical writers, shows that it must have long existed, and that it was in all probability one of the most ancient modes of manual interference. When the child was thought to be dead perforation or embryotomy was at once resorted to, and the impossibility of bringing down the head in many cases must have occasioned the frequent destruction of infants whose life might have been preserved. The accuracy with which many of the diseases of women are described in the Hippocratic writings, makes it the more to be regretted that their authors had such scanty opportunities for cultivating practical midwifery.

Many of Aristotle's observations on the physiology of pregnancy and labour are very valuable, especially his researches into these processes in animals. His labours, however, excited no influence on the practice of midwifery, which still continued exclusively in the hands of women.

The condition of the art among the Romans was by no means better than among the Greeks. The social state of a people engaged, as the Romans were, in almost constant war, affords but little encouragement to the cultivation of medicine. Accordingly, the Romans, during the republic, possessed but little knowledge of the art beyond the elements of a rude surgery, while they endeavoured to make up for their ignorance by the grossest superstition. Gods almost numberless presided over pregnancy, labour, and the education of children. Lucina was implored under various names, and worshipped, now as Juno, now as Diana. Prosa and Postverta superintended childbirth; the former when its course was natural, the latter when the presentation was unfavorable. The Dii Nixii and the goddess Numeria strengthened the throes of labour, and hastened delivery; Pilumnus, Intercidona, and Deverra watched over the safety of the woman and her new-born babe; Carna protected children in the cradle; Rumina presided over the process of suckling; Educa and Potina took care of their food in general; Cunina watched by the cradle; Ossipaga moulded the bones; Vaticanus waited on the cries of children; and Fabulinus helped them in their first attemps to speak. But we will cease enumerating these gods, the long catalogue of whom is not nearly exhausted. Women were the sole practitioners of midwifery in Rome, and were either wholly ignorant, or in latter ages derived a scanty instruction from Greek physicians or midwives who settled in Italy. Some advances had nevertheless been made since the time of Hippocrates, for we find Celsus advocating version by the feet, a practice, which, if it had been followed, would have saved the lives of many mothers and infants who fell victims to the Hippocratic mode of attempting to bring down the head to the brim of the pelvis. Celsus indeed did not recommend the practice with a view of saving the child, for we find that in the cases to which he refers, the child was supposed to be dead, and the preservation of the mother's life was the only point which claimed attention.

Between Celsus and Galen, the names of many pass under review, almost all of whom contributed their quota to the advance of the theory of the art, though they did but little towards improving its practice. Galen indeed, who enriched theoretical midwifery more than any of his predecessors, did almost nothing for its practice, in which he is inferior to Celsus or Hippocrates. With Galen, this period terminates, and we come to a third which offers but little worth recording. (pp. 70-179.)

*Third period.* This extends from the decline of science to the cultivation of medicine by the Arabs.

This was a time of dark superstition, when faith in charms and amulets and magic prevailed universally among the vulgar, and a blind copying of Galen stood in the stead of scientific investigation. Midwifery continued, as might be expected, the undisputed field of ignorance and charlatanry. The help of the physician was sought only in desperate cases, when operations were performed which always destroyed the child and frequently endangered the life of the mother. One cause of the slight value attached to the life of the child was doubtless the opinion that, while in the womb, it is not endowed with a soul; against which notion and the consequent practice Tertullian loudly exclaimed.

Ætius of Amida was the best writer of the day; a diligent compiler who borrowed from many whose names only are known to us, and among others from Philumenos. One great merit of Philumenos was his recommendation of the operation of turning, which in after years fell quite into disuse till its employment was revived by Ambrose Paré. The rules given by Philumenos for the management of the placenta, are likewise extremely judicious, and most of them are adhered to in the practice of the present day. The last person of any note in this period was Paul, of Ægina, whose investigations into the diseases of women are very valuable. In matters of practical midwifery, however, and especially as respects the operation of turning, we find that opinion had retrograded during the century which had elapsed since Ætius. (pp. 181-240.)

*Fourth period.* This is that in which the cultivation of medicine was confined to the Arabs; an age in which the advance made was small, though time was occupied, not altogether uselessly, in examining and arranging the labours of by-gone years. The barbarous operations of midwifery were still retained, but attempts were made to lay down laws for their employment. The first author during this period was Serapion, in whose practice we find full proof of the decline of midwifery since the days of Ætius. He forbids the extraction of the child by the feet, and recommends in different labours that the feet should be amputated, with the view of pushing back the child, and then bringing down the head to the brim of the pelvis. Rhazes, who lived about half a century later, notwithstanding his great reputation, has scarcely in any respect improved upon the maxims of his predecessor. One point which cannot escape us in reading the works of those days is the utter ignorance which existed respecting the pelvis. A difficult labour was supposed to be often produced by a small uterus or a narrow vagina, but no conception was formed of hinderances to parturition arising from the state of the bony parts. Instances of this opinion are adduced from Serapion and Ali Abbas. Avicenna is the best specimen we could find of the spirit of the age, and the obstetric remarks in his canon of medicine contain a strange mixture of good and evil, and show that the bloody and brutal mutilations of the child was still in common practice. For a knowledge of many of the obstetric instruments of those days we are indebted to Abulkasem, who wrote a treatise on midwifery, and illustrated it with drawings.

From the time of Abulkasem to the commencement of the fifth period we meet with few names, and none of them those of men who did much for the improvement of obstetricy. (pp. 241-302.)

*Fifth period.* The post-Arabian era is made by Von Siebold to extend to the publication by Eucharius Roesslin, of the first printed book on midwifery. It was a time of advancement for medical science generally, but illustrated by few men who devoted themselves to obstetric subjects. The circumstance of medicine being in the hands of the monks necessarily confined the practice of midwifery still to women. The revival of anatomical study, however, overthrew many fallacies, and paved the way for the improvements which commenced with the beginning of the sixteenth century. (pp. 303-360.)

We look anxiously for the appearance of the second volume, which is to introduce us to these better days; but, in the mean while, our readers will thank us for having called their attention to the book. It contains much information of general interest; and to any who are engaged in teaching midwifery it is absolutely indispensable. Its possession will spare them many an hour that they might otherwise spend in turning over the pages of old and forgotten tomes, from which, after all their labour, they would not obtain nearly so much information respecting the midwifery of the ancients as the elegant work of Professor von Siebold will convey to them in half an hour.

---

